# *TPH1* A218C polymorphism and temperament in major depression

**DOI:** 10.1186/1471-244X-13-118

**Published:** 2013-04-18

**Authors:** Kadri Andre, Olli Kampman, Merja Viikki, Ari Illi, Eija Setälä-Soikkeli, Outi Poutanen, Nina Mononen, Esa Leinonen, Terho Lehtimäki

**Affiliations:** 1 Mental Health Center, City of Helsinki, Finland; 2 School of Medicine, University of Tampere, Tampere FI-33014, Finland; 3 Department of Psychiatry, Tampere University Hospital, Tampere, Finland; 4 Department of Psychiatry, Kanta-Häme Central Hospital, Hämeenlinna, Finland; 5 Department of Psychiatry, Seinäjoki Hospital District, Seinäjoki, Finland; 6 Tampere Mental Health Centre, Tampere, Finland; 7 Department of Psychiatry, Satakunta Hospital District, Harjavalta, Finland; 8 Centre for Laboratory Medicine, Tampere University Hospital and Department of Clinical Chemistry, University of Tampere, School of Medicine, Tampere, Finland

**Keywords:** Depressive disorder, Temperament, TCI, Antidepressive agents, Treatment response, TPH1

## Abstract

**Background:**

In major depression, one of the candidate genes possibly affecting the risk and severity of symptoms has been found to be tryptophan hydroxylase (*TPH1*). Variation in treatment response to antidepressive agents according to *TPH1* genotype has also been found in several studies. However, the relationship between temperament and *TPH1* genotype in major depression is poorly understood, as only one study has been published so far. There are no earlier studies on the interaction between temperament traits, antidepressive medication response and *TPH1* genotype. This interaction was studied in 97 subjects with major depression treated for six weeks with selective serotonine reuptake inhibitors.

**Methods:**

Temperament dimensions Harm Avoidance (HA), Novelty Seeking (NS), Reward Dependence (RD) and Persistence (P) scores at baseline (1) and endpoint (2) were rated with the Temperament and Character Inventory (TCI) and compared between *TPH1* A218C genotypes. Multivariate analysis of co-variance (MANCOVA) was used to analyze the interaction between the *TPH1* genotype, treatment response and the different temperament dimensions at baseline and endpoint. In the analysis model, treatment response was used as a covariate and *TPH1* genotype as a factor. A post hoc analysis for an interaction between remission status and *TPH1* A218C genotype at endpoint HA level was also performed.

**Results:**

The number of *TPH1* A-alleles was associated with increasing levels in NS1 and NS2 scores and decreasing levels in HA1 and HA2 scores between *TPH1* A218C genotypes. In the MANCOVA model, *TPH1* genotype and treatment response had an interactive effect on both HA1 and HA2 scores, and to a lesser degree on NS2 scores. Additionally, an interaction between remission status and *TPH1* A218C genotype was found to be associated with endpoint HA score, with a more marked effect of the interaction between CC genotype and remission status compared to A-allele carriers.

**Conclusions:**

Our results suggest that in acute depression *TPH1* A218C polymorphism and specifically the CC genotype together with the information on remission or treatment response differentiates between different temperament profiles and their changes.

## Background

Tryptophan hydroxylase (TPH) is a rate-limiting enzyme involved in the synthesis of neurotransmitter serotonin (5-HT) and therefore determines the levels of 5-HT being released [1]. *TPH1* A218C polymorphism has been suggested to have an impact on depressive disorders and suicidal behavior [2]. According to a meta-analysis the overall response rate to antidepressive treatment was found to be better for CC-genotype than for A-allele carriers. However, only two of the studies included showed a significantly better response for A-carriers [3]. By contrast, in a study where *TPH1* CC-genotype was found to occur more commonly among patients with major depression, the same genotype was associated both with the severity of the disease and with lower probability of achieving remission [4].

So far only one study has been published on temperament traits and *TPH1* A218C polymorphism in major depression; it reports a negative result between HA or NS scores and *TPH1* genotypes [5]. The C allele of A218C has been reported to be more common among nonorganic and nonpsychotic inpatients with impulsive behavioral traits [6]. Higher harm avoidance scores and more severe binge eating behavior were observed among bulimic women with AA genotype [7]. High scores on HA were found with AA genotype of *TPH1* and a hostile childhood environment in a study on healthy adults exploring the role of earlier socioeconomic factors [8]. In a sample of healthy Chinese subjects a trend towards higher novelty seeking scores was found among men with CC genotype [9].

The TPH enzyme occurs in two different isoforms [10]. *TPH1* gene is located on chromosome 11p15.3-p14 and is expressed in the gut, spleen, thymus, but also in the pineal gland and in the pituary [11]*TPH1* gene variants may have an influence on the level of serotonin metabolites and their functionality, because lower cerebrospinal fluid 5-hydroxyindoleacetic acid (5-HIAA) levels were found in men with the *TPH1* A218C A-allele, but not in women [12]. However, another study on the association of A218C with serotonin metabolites resulted in negative findings [13]. *TPH1* A218C polymorphism has been observed to have an effect on amygdala activity; subjects with the A allele showed greater brain activity in the bilateral amygdala under sad vs. neutral condition than subjects homozygous for the C allele [14].

The TCI is a questionnaire which distinguishes four temperament dimensions, namely Harm Avoidance (HA), Novelty Seeking (NS), Reward Dependence (RD) and Persistence (P) [15]. Several studies, including our earlier study [16], report negative outcomes concerning the association between *TPH1* genotype, depression and its treatment response [5,17]. In a number of studies temperament traits, and most importantly HA, have shown an impact on recovery from depression [18].

We investigated the interaction between *TPH1* A218C polymorphism, SSRI treatment response and temperamental traits assessed by the Temperament and Character Inventory (TCI) in a clinical sample of subjects with major depression. The primary aim of the study was to analyze the relationship between temperament dimensions and *TPH1* genotype, with treatment response as a secondary aim. The study hypotheses included 1) relationship between *TPH1* genotype and temperament dimensions and 2) interactive effect of *TPH1* genotype and antidepressive response on temperament dimensions.

## Methods

### Study subjects and clinical intervention

The patients were recruited for a pharmacogenetic study on depression during the time period from September 2002 to December 2006. The population consisted of 97 individuals 19-72 years of age (41 males and 56 females, age mean [SD] 40.5 [14.1] years). All subjects were patients in secondary outpatient services in Pirkanmaa Hospital District, Finland (total catchment population approximately 300,000). Study inclusion criteria were: 1) current episode of major depressive disorder according to DSM-IV criteria and 2) a Montgomery Åsberg Depression Rating Scale (MADRS) [19] score of at least 20. Treatment response was taken to be a reduction of at least 50% in MADRS score during follow-up, and remission as MADRS scores of 7 or less. The dosage of the study medication was adjusted according to the clinical response, which was checked after three weeks during a short study visit. Medication adherence was self-monitored by keeping a paper-and-pencil medication diary. At least 80% adherence rate was considered adequate treatment.

The clinical researchers interviewed each patient before the initial assessment for the study. Patients with major somatic diseases and those with medications potentially causing depression were excluded, likewise patients with bipolar disorder, schizophrenia, severe personality disorders or disorders related to substance abuse. Patients had to have been free of antidepressive medications for the past three months, and mood stabilizing or anti-psychotic medications were not allowed. Anxiolytics and hypnotics in minor doses were permitted at the early stage of the study.

Patients completed the 107-item TCI temperament questionnaire (version IX) at the initial assessment of the study and after six weeks of follow-up. All subjects received SSRI medication, citalopram, fluoxetine or paroxetine (the three most frequently prescribed SSRIs in Finland at the time of the study). All patients gave written informed consent and the local ethics committee approved the study protocol.

### DNA extraction and *TPH1* A218C genotyping

Genomic DNA was extracted from peripheral blood leukocytes using QIAamp®DNA Blood Minikit and automated biorobot M48 extraction (Qiagen, Hilden, Germany). *TPH1* A779C (rs1799913) was genotyped with Taqman®SNP Genotyping Assay C_2645661_10 and *TPH1 A218C* (rs 1800532) with a custom Taqman Assay (Applied Biosystems). Information on oligos and PCR protocol are available from the corresponding author.

*TPH1 A218C* and A779C polymorphisms were in complete linkage disequilibrium in the present study subjects. Thus, to avoid repetition, only the results concerning A218C polymorphisms are reported here.

### Statistical methods

**Figure 1:**
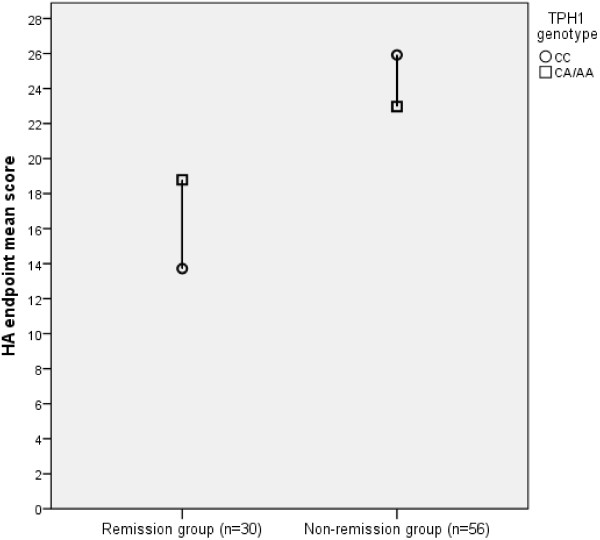
**Mean harm avoidance endpoint scores in remitters and non-remitters according to TPH1 A-allele carrying status.** The interaction of TPH1-genotype (CC vs. CA/AA) and the remission status significantly explained the HA endpoint score (p < 0.001, ηp^2^ = 0.209, power = 0.98, GLM univariate model).

Temperament dimension HA, NS, RD and P scores at baseline (1) and endpoint (2) during antidepressive treatment were analyzed between *TPH1* genotypes with ANOVA including Bonferroni correction. Before the ANOVA analyses, the normality of distributions was checked in each temperament/genotype subgroup, and these showed normal distributions. Multivariate analysis of co-variance (MANCOVA) was used to explain the different temperament dimensions (HA, NS, RD and P) at baseline and endpoint. In the model, treatment response was used as a covariate and *TPH1* genotype as a factor. Finally, as a post-hoc analysis, the HA endpoint score was predicted by the interaction of *TPH1* A-allele carrying and remission status with a GLM univariate model (Figure 1).

In power analysis the temperament score limits were determined in such a way that we were able to detect with a statistical power of ≥0.8 between different *TPH1* genotype groups. They were 3.4, 3.9 and 4.4 between CC/CA (n = 73), CC/AA (n = 56), and CA/AA (n = 45) respectively.

## Results

Frequencies of baseline and endpoint medications, distributions of corresponding temperament traits, and depressive symptoms are given in Table 1. In keeping with the primary aim of the study in comparisons with TCI temperament dimensions and *TPH1* genotypes, we observed a linearly increasing change between *TPH1* CC (n = 34/31), CA (n = 47/42) and AA (n = 16/14) genotypes in NS1 and NS2 scores (NS1: CC 17.2 ± 7.4, CA 19.8 ± 7.1, AA 23.6 ± 7.2, p = 0.016; NS2: CC 18.7 ± 6.7, CA 20.6 ± 7.7, AA 26.4 ± 5.7, p = 0.005). A similar, but decreasing change was found in HA1 between *TPH1* genotypes and to a lesser degree in HA2 (HA1: CC 25.4 ± 6.6, CA 23.8 ± 6.5, AA 19.8 ± 7.6, p = 0.027; HA2: CC 23.2 ± 8.5, CA 22.3 ± 7.5, AA 17.2 ± 6.9, p = 0.055). In P1, a trend level difference was found between *TPH1* genotypes (CC 4.0 ± 2.2, CA 4.9 ± 2.0, AA 3.7 ± 1.6, p = 0.058). In P2, RD1 and RD2 no significant differences in temperament scores between genotypes were found.

**Table 1:** Frequencies of medications and distributions of temperament traits and depressive symptoms during the study

**Variable**	**Baseline (n = 97)**		**Endpoint (6 weeks, n = 87)**	
**Antidepressive medication**	**N**	**%**	**N**	**%**
Citalopram	50	51.5	44	50.6
Paroxetine	12	12.4	11	12.6
Fluoxetine	35	36.1	32	36.8
Use of hypnotics	32	33.0		
Use of anxiolytics	22	22.7		
Temperament scores	mean	SD	mean*	SD
NS	19.5	7.5	20.8	7.4
HA	23.7	6.9	21.8	7.9
RD	15.5	3.9	15.9	3.7
P	4.3	2.0	4.3	2.0
MADRS score	26.9	5.6	12.2	8.2
Antidepressant dose, mg	19.8	2.7	22.3	6.5

*n = 86.

*NS* novelty seeking, *HA* harm avoidance, *RD* reward dependence, *P* persistence.

In keeping with the secondary aim of the study a MANCOVA analysis was performed. The results of the MANCOVA model are presented in Table 2. The GLM univariate model with HA2 as a target variable and *TPH1* A allele carrying and remission status as explanatory variables showed an interactive effect (p < 0.001, ηp^2^ = 0.209, power = 0.98; Figure 1). In this model, the CC genotype when compared to A-carriers had a more marked interactive effect with remission status on the post-treatment HA level.

**Table 2:** **Results of the MANCOVA model, in which all temperament dimensions (HA, NS, RD and P) at baseline (1) and at endpoint (2) were used as target variables and *TPH1 *genotype as a factor and MADRS score change as a covariate**

	**Complete model**			***TPH1 *genotype**		**ΔMADRS**	
**Target variable**	**ηp**^**2**^	**p**	**Power**	**ηp**^**2**^	**p**	**ηp**^**2**^	**p**
HA1	0.186*	0.001	0.96	0.120**	0.005	0.078	0.01
HA2	0.252	<0.001	1.00	0.079	0.034	0.196	<0.001
NS1	0.139	<0.001	0.86	0.120	0.005	0.02	0.20
NS2	0.199	<0.001	0.97	0.127	0.004	0.087	0.006
RD1	0.071	0.11	0.51				
RD2	0.042	0.32	0.31				
P1	0.082	0.07	0.59				
P2	0.112	0.02	0.76	0.33	0.027	0.091	0.005

* ηp^2^ = explanatory proportion of the complete model for the target variable.

** ηp^2^ = explanatory proportion of the single factor or covariate for the target variable.

## Discussion

This is the first study to investigate the interaction between temperament traits, antidepressant treatment response and *TPH1* A218C genotype. Associations between *TPH1* genotype and temperament dimensions were studied in patients with major depressive disorder and linear changes were found at both baseline and endpoint. Subjects with the C-allele of A218C scored higher on HA and lower on NS. HA scores were higher at baseline than at endpoint and NS scores increased from baseline to endpoint. In multivariate analysis of covariance (MANCOVA) *TPH1* genotype was used as a factor and MADRS scores as a covariate, since depressive symptoms have been found to modify temperamental traits and, on the other hand, temperament has an impact on recovery from depression [18]. HA and NS scores at baseline were analyzed as temperament profile itself can reflect the biological subtype of depression and be associated with the clinical response. Treatment response has been found to affect HA and NS scores at endpoint [20]. A possible interaction of *TPH1* genotype and temperament dimensions was not separately analyzed in treatment response due to the obvious associations detected between HA and NS dimensions and *TPH1* genotypes. Additionally, an interaction of remission status and most markedly with *TPH1* CC genotype was found to be associated with endpoint HA score. According to this analysis, the endpoint HA score variation was greater with the CC genotype than with the AG/AA genotypes when the remission and non-remission groups were compared. This finding may indicate the CC genotype being associated more with traits related to risk of depression, including the tendency to harm avoidance, than with the depressive state itself. Earlier studies, including one meta-analysis, have reported contradictory results on the relationship between *TPH1* genotype and treatment response in major depression [2,4]. In a population based study no association was found between *TPH1* genotype and TCI temperament dimensions [21]. Both the depressive disorder among our patients and the different ethnic background may explain the discrepancy in the results. It was therefore important to study the possible interactive effect of genotype and treatment response on temperament dimensions. As the impact of genotype alone is likely very small it can be hypothesized to be connected with the depressive trait rather than the clinical state.

According to present results *TPH1* genotype and MADRS scores explained 14-25 percent of the changes in HA and NS scores from baseline to endpoint. *TPH1* genotype explained about half of the variance within the linear model. HA scores at baseline were explained mostly by *TPH1* genotype (number of A alleles related with lower HA), and treatment response (better response related with lower HA) had less impact. HA scores at endpoint were explained to a greater extent by treatment response and less by genotype. The prospective setting could be considered as a strength of this study. At follow-up visits the adequacy of antidepressive treatment was monitored by adherence diaries, symptom evaluations and dosage adjustments, if necessary. The limitations of the study are the relatively small patient sample, in spite of a satisfactory statistical power in separate analysis. Also, the study setting was focused primarily on the acute treatment response. No structured interviews were conducted, and thus no evaluation of axis II disorders was available. It is possible that in some patients personality disorders had an impact on treatment response.

These results suggest that C allele of *TPH1* is associated with differences in temperament profile. High HA among C allele carriers may lead to general avoidance behavior and susceptibility to depression. There is one earlier study reporting no association between temperament dimensions and *TPH1* genotype [5], but in this sample most of the patients had a diagnosis of bipolar disorder. The different findings likely reflect the different genetic backgrounds of bipolar and unipolar depressive disorders.

## Conclusion

According to the present study, *TPH1* A218C genotype differentiates between temperament profiles and changes therein in acute major depression, which is supported by genotype-specific differences found in HA and NS scores at baseline and endpoint during antidepressive treatment. In the comparison of remitters and non-remitters, CC genotype had the most marked interactive effect on HA endpoint scores. Given the clinical state of major depression and the underlying risk traits, it seems that despite the achievement of remission the impact of depression risk traits differs depending on the *TPH1* genotype.

## Competing interests

All authors report no competing interest regarding the submitted study.

## Authors’ contributions

Authors AI, OK and EL designed the study and wrote the protocol. Authors OK, MV, AI, ESS, OP and EL participated in patient recruitment. Authors NM and TL carried out the molecular genetic studies and participated in the sequence alignment. Authors KA and OK managed the literature searches and undertook the statistical analysis. Author KA wrote the first draft of the manuscript. All authors read and have approved the final manuscript.

## Pre-publication history

The pre-publication history for this paper can be accessed here:

http://www.biomedcentral.com/1471-244X/13/118/prepub
